# Integral Light-Harvesting Complex Expression In *Symbiodinium* Within The Coral *Acropora aspera* Under Thermal Stress

**DOI:** 10.1038/srep25081

**Published:** 2016-04-27

**Authors:** Sarah L. Gierz, Benjamin R. Gordon, William Leggat

**Affiliations:** 1 College of Public Health, Medical and Veterinary Sciences, James Cook University, Townsville, 4811, Australia; 2 Comparative Genomics Centre, James Cook University, Townsville, 4811, Australia; 3 ARC Centre of Excellence for Coral Reef Studies, James Cook University, Townsville, 4811, Australia

## Abstract

Coral reef success is largely dependent on the symbiosis between coral hosts and dinoflagellate symbionts belonging to the genus *Symbiodinium*. Elevated temperatures can result in the expulsion of *Symbiodinium* or loss of their photosynthetic pigments and is known as coral bleaching. It has been postulated that the expression of light-harvesting protein complexes (LHCs), which bind chlorophylls (chl) and carotenoids, are important in photobleaching. This study explored the effect a sixteen-day thermal stress (increasing daily from 25–34 °C) on integral LHC (chlorophyll *a*-chlorophyll *c*_*2*_-peridinin protein complex (*acpPC*)) gene expression in *Symbiodinium* within the coral *Acropora aspera.* Thermal stress leads to a decrease in *Symbiodinium* photosynthetic efficiency by day eight, while symbiont density was significantly lower on day sixteen. Over this time period, the gene expression of five *Symbiodinium* *acpPC* genes was quantified. Three *acpPC* genes exhibited up-regulated expression when corals were exposed to temperatures above 31.5 °C (*acpPCSym_1:1*, day sixteen; *acpPCSym_15*, day twelve; and ^acpPCSym_18^, day ten and day sixteen). In contrast, the expression of *acpPCSym_5:1* and *acpPCSym_10:1* was unchanged throughout the experiment. Interestingly, the three *acpPC* genes with increased expression cluster together in a phylogenetic analysis of light-harvesting complexes.

Photosynthetic eukaryotic dinoflagellates belonging to the genus *Symbiodinium* form symbiotic relationships with a variety of marine taxa. Endosymbiotic associations observed between these photosynthetic dinoflagellates and corals are generally classed as mutualistic, as both the host and symbiont benefit from the relationship^1^,^2^. The photosynthetic symbiont acquires nutrients such as inorganic carbon, nitrogen and phosphate from host cells^3^, in turn symbionts then provide up to 90% of the energy required by corals to grow and reproduce. These relationships between scleractinian corals and *Symbiodinium* are critical for the proliferation of reefs supporting diverse marine ecosystems. Coral bleaching, the loss of photosynthetic pigments or endosymbionts from host cells occurs under stress conditions such as elevated sea temperatures of only a few degrees above long-term maxima^4^,^5^. While ocean temperature fluctuations occur on a daily basis, the mean sea surface temperature is predicted to rise by approximately 1–2 °C over the next century and is expected to lead to more mass bleaching events^6^,^7^.

Experimentation on *Symbiodinium* and the coral holobiont has focussed on many environmental factors implicated in the onset of coral bleaching including elevated seawater temperatures, eutrophication and disease. The effect of high sea-surface temperatures have been a key focus due to mass coral bleaching events (~42% GBR reefs bleached in 1998 and ~54% reefs bleached in 2002^8^), attributed to global climate change^7^ with the 1998 bleaching event coinciding with an El Niño Southern Oscillation event^4^,^9^.

Differential thermal stress sensitivity is observed across the diverse *Symbiodinium* species complex, with both heat tolerant and heat sensitive species observed within the same clade^10^. Differences in photoinhibition sensitivity have either been acquired independently by thermally tolerant types or have been acquired in the common ancestor of all *Symbiodinium* types and since lost in thermally sensitive species^10^. Elucidation of sites of thermal sensitivity within *Symbiodinium* has focussed on potential points where damage results in a decrease in photosynthetic efficiency^11^,^12^. These potential points include damage to the D1 protein of photosystem II (PSII)^12^, inhibition of the *de novo* synthesis of the D1 protein^13^, the enzyme Rubisco^14^, thylakoid membrane integrity^10^, the carbon concentrating mechanism^15^ and LHCs^16^. However, none of these sites have conclusively been demonstrated as the initial site of thermal damage.

LHCs, also called antenna proteins, are found in photoautotrophic organisms and are an array of protein, chl and accessory pigment molecules with roles in light-harvesting and photoprotection^17^,^18^. In *Symbiodinium* the light-harvesting system can be divided into two associated complexes, the highly conserved core LHCs, and variable periphery LHCs^19^. Studies of green plant LHCs have elucidated structural information, which has further improved the understanding of light capture and transfer, and the arrangement of peripheral LHCs in photosynthetic eukaryotes^18^,^20^. Peripheral LHCs may be categorized within the large gene super-family based on associated pigments into three related groups of pigment binding proteins^21^,^22^. The first group binds chl *a* and *b*, the second binds chl *a* and *c* and the third group binds chl *a* and phycobilins. The chl *a/c* lineage LHCs are additionally divided into the fucoxanthin-chl *a/c* and peridinin–chl *a/c* (PCP) complexes, which are found in dinoflagellates^21^,^22^,^23^.

In *Symbiodinium* two types of peripheral LHCs are found, PCP and acpPC^19^. Dinoflagellate PCPs share no sequence similarity with other known LHCs and are water soluble complexes found on the luminal periphery of thylakoid membranes^24^. In contrast, dinoflagellate acpPCs are integral thylakoid membrane complexes that share sequence similarity with the chl *a*/*c* subfamily of LHCs^24^. Further, characterisation of *Symbiodinium* C3 *acpPCs* and *Symbiodinium* A1.1 LHCs cluster sequences with three clades within the chl *a*/*c* binding LHC family, indicating high diversity of these proteins within species^21^. Dinoflagellate and *Symbiodinium* PCPs are well studied due to their unique features^25^,^26^,^27^,^28^. In *Symbiodinium* the LHCs have been shown to decrease energy transfer and dissociate from the photosystem reaction centres following photoinhibition in order to protect cells during stress events^12^,^29^,^30^,^31^,^32^. Decreasing the number of peripheral LHCs available to absorb and transfer energy is a proposed photoprotection mechanism, as this reduces the amount of light reaching the reaction centres and limits the risk of possible photodamage to the D1 reaction centre proteins^30^. Further, kinetic studies have revealed that LHC proteins disassociate and reattach to thylakoid membranes under increased light levels to ameliorate stress^32^. However, few studies have examined the effect of thermal stress on *Symbiodinium* acpPC expression^16^,^32^.

Targeted studies of *Symbiodinium* transcript levels have shown that changes in gene expression occur on a relatively small scale. Quantitative-PCR has been used to determine changes in a variety of genes of interest related to stress response^33^,^34^,^35^,^36^. The validation of housekeeping genes for use in *Symbiodinium* has allowed for a reference to be established in order to determine differential gene expression under various conditions^36^,^37^. Although significant changes have been observed in *Symbiodinium* physiology and large fold changes in host gene expression have been recorded in cells exposed to stress, changes in *Symbiodinium* gene expression occur at a far smaller scale (±<5-fold)^33^,^34^,^35^.

This study focussed on the response of *acpPC* genes in *Symbiodinium* cells during a prolonged thermal stress event (sixteen days) in *Acropora aspera.* We find that the response of five *acpPC* genes in *Symbiodinium* under stress varies across a LHC phylogeny. This is the first study to investigate the effect of thermal stress on gene expression patterns in the LHC superfamily within a coral host.

## Results

### *Symbiodinium* density

**Figure 1:**
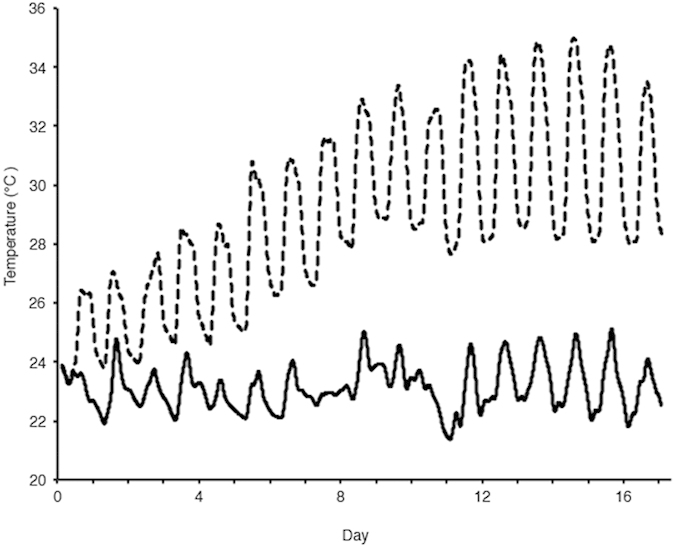
Temperature of ambient (solid line) and heated treatment (dashed line) during the sixteen-day thermal experiment. Values represent the average of 4 replicate tanks at control and treatment temperatures.

**Figure 2:**
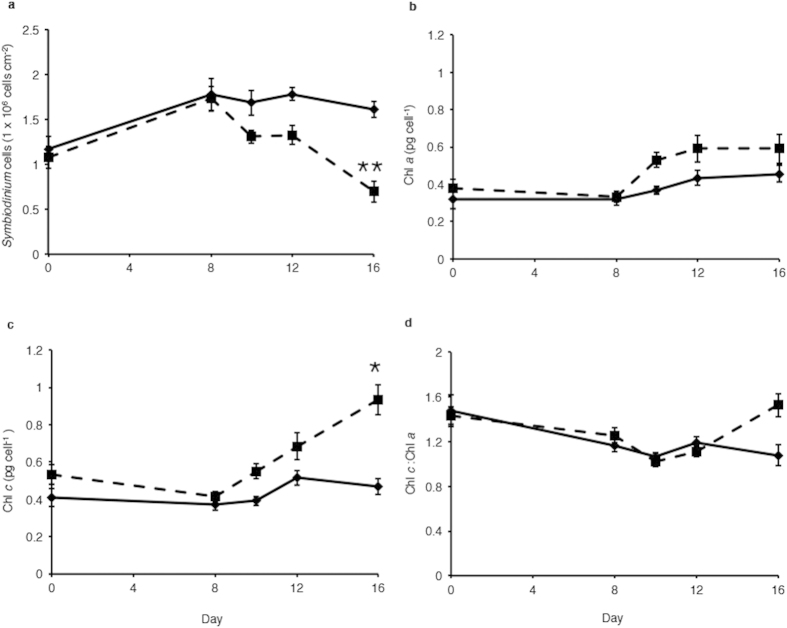
(**a)** *Symbiodinium* cell density per cm^2^ in *A. aspera* (**b**) *Symbiodinium* chl *a* pigment concentrations in *A. aspera* (**c**) *Symbiodinium* chl *c* pigment concentrations in *A. aspera*. (**d**) Ratio of chl *c* to chl *a* per *Symbiodinium* cell in *A. aspera* nubbins. *A. aspera* nubbins subjected to control conditions (solid line) and heated treatment (dashed line). *Error bars* represent ± s.e.m., *n* = 9–12, some *error bars* obscured by data point markers. The statistical difference (*post hoc* sequential Bonferroni analysis) between treatment and control is indicated as *p < 0.05 or **p < 0.01.

Over a period of sixteen days, branches of *A. aspera* were exposed to temperature increasing from ambient levels (~25 °C) to a bleaching temperature of ~34 °C (Fig. 1). As has previously been found, this temperature increase led to a significant decrease in *Symbiodinium* cell densities (*p* < 0.001) over the course of the experiment, with average densities of 6.0 × 10^5^ cells cm^−2^ in the treatment corals on day sixteen, compared to 1.6 × 10^6^ cells cm^−2^ in the control corals on the same day (Fig. 2a).

### Chl pigment content

Chl *a* content per *Symbiodinium* cell increased over the experimental period in the heated treatments from day eight of the experimental period (Fig. 2b). Analysis of the chl *a* content found that there were significant differences between day (*p* < 0.001, df = 5) and treatment (*p* < 0.05, df = 1) but not in the interaction treatment × day (*p* > 0.05, df = 5) (Fig. 2b). Similarly chl *c* content per *Symbiodinium* cell increased from day ten onwards in the experiment period (Fig. 2c). Analysis of the chl *c* content found that there were significant differences between day (*p* < 0.01, df = 5) and treatment (*p* < 0.01, df = 1) but not in the interaction treatment × day (*p* > 0.05, df = 5) (Fig. 2c). The ratio of chl *c* to chl *a* was unchanged between control and treatment conditions throughout the experiment (Fig. 2d).

### Chl fluorescence and photosynthetic efficiency

**Figure 3:**
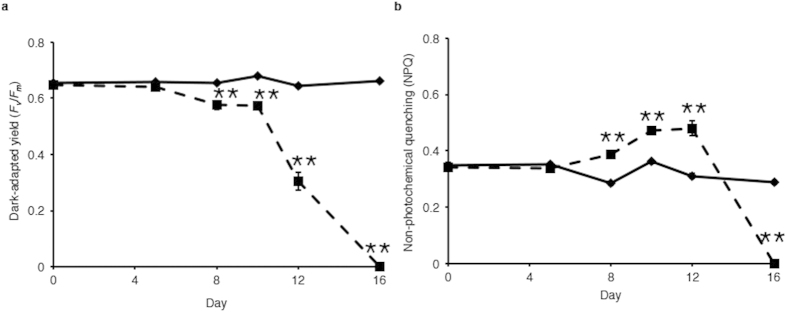
(**a**) *Symbiodinium F*_*v*_*/F*_*m*_ within *A. aspera* during the experiment. (**b**) *Symbiodinium* NPQ within *A. aspera* at the last point of the induction phase during the Imaging-PAM analysis. *A. aspera* nubbins exposed to control conditions (solid line) and heated treatment (dashed line). Values represent average obtained from twelve biological replicates across four replicate tanks. *Error bars* represent ± s.e.m., *n* = 12, some *error bars* obscured by data point markers. The statistical difference (*post hoc* sequential Bonferroni analysis) between treatment and control is indicated as *p < 0.05 or **p < 0.01.

Maximum quantum yield of photosynthesis (*F*_*v*_*/F*_*m*_) was measured following sunset during the experiment. For corals maintained at control temperatures, *F*_*v*_*/F*_*m*_ was between 0.602 and 0.692 (average 0.657) (Fig. 3a). Analysis of *F*_*v*_*/F*_*m*_ found that there was a significant effect between day and treatment (*p* < 0.001, df = 5) and differences between day (*p* < 0.001, df = 5) and treatment (*p* < 0.001, df = 1) (Fig. 3a). A sequential Bonferroni *post hoc* analysis found that *F*_*v*_*/F*_*m*_ decreased in the heated treatment on days eight, ten, twelve and sixteen of the experiment compared to controls from the same days (*p* < 0.01) (Fig. 3a).

Non-photochemical quenching (NPQ) was also measured over the course of the experiment. As with *F*_*v*_*/F*_*m*_, there were significant interaction effects between treatment × day (*p* < 0.001, df = 5), and between day (*p* < 0.001, df = 5) and treatment (*p* < 0.05, df = 1) (Fig. 3b). *Post hoc* analysis demonstrated NPQ was significantly increased by heating on days ten, twelve and fourteen before declining to zero on the final day of the experiment (Fig. 3b).

### Gene expression under thermal stress

**Figure 4:**
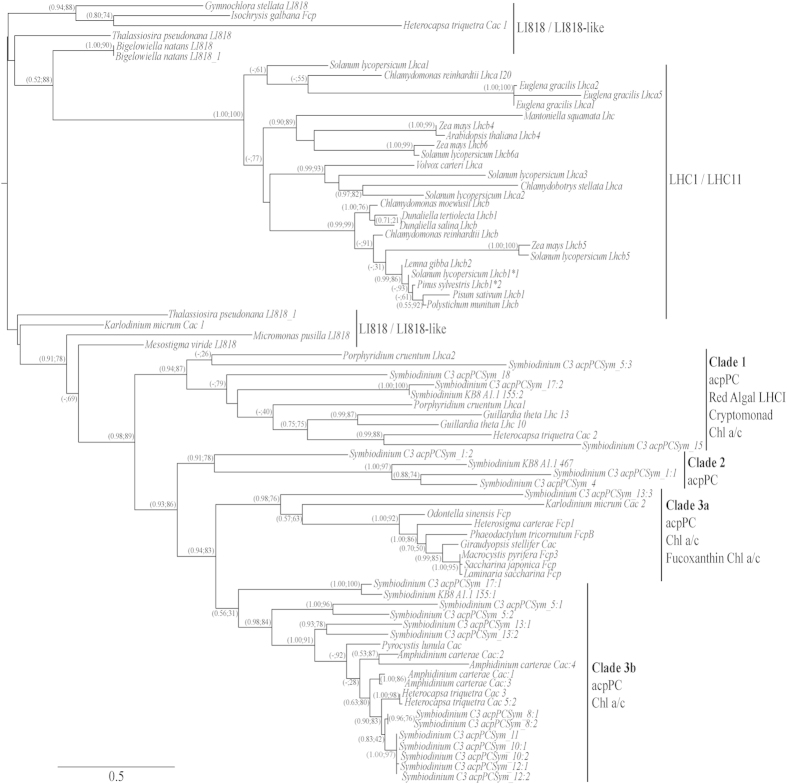
Phylogenetic analysis of with LHCs from chl *a/b* and chl *a/c* containing organisms. Chl *a/b* binding protein complexes cluster together while the chl *a/c* binding protein complexes form a second cluster. *Symbiodinium* sp. C3 *acpPC* sequences and *Symbiodinium* type A1.1 LHCs are found throughout the four clades (Clade 1-3b) of the chl *a/c* binding protein complexes. Reproduced from Boldt *et al.*^21^.

**Figure 5:**
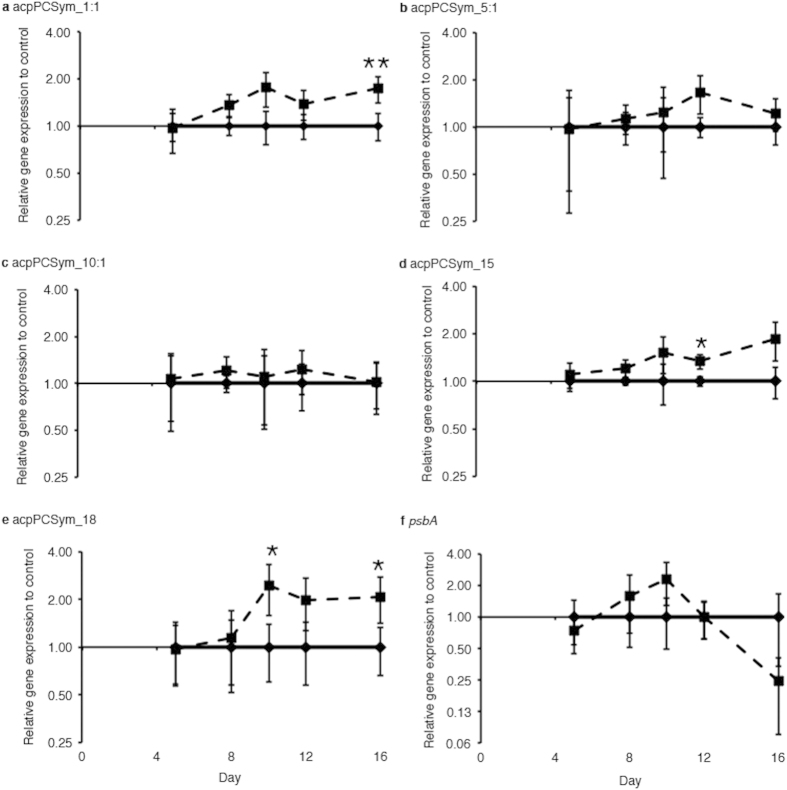
Relative expression of *Symbiodinium* genes of interest when exposed to prolonged thermal stress. Values expressed as relative expression of treatment (dashed line) to control (solid line) for each time point: (**a**) *acpPCSym_1:1*, (**b**) *acpPCSym_5:1*, (**c**) *acpPCSym_10:1*, (**d**) *acpPCSym_15*, (**e**) *acpPCSym_18* and (**f**) *psbA*. *Error bars* represent ± s. e. m., n = 4–10, some *error bars* obscured by data point markers. The statistical differences (*post hoc* sequential Bonferroni analysis) between treatment transcript abundance and control is indicated as *p < 0.05 or **p < 0.01.

The expression of five *acpPC* genes (*acpPCSym_1:1*, *acpPCSym_5:1*, *acpPCSym_10:1*, acpPCSym_15 and *acpPCSym_18*) from three distinct LHC clades (Fig. 4) was determined. Over the course of the experiment three *acpPC* genes were found to have significant increases in gene expression, *acpPCSym_1:1* on day sixteen (1.74-fold, *p* = 0.001), *acpPCSym_15* on day twelve (1.33-fold, *p* = 0.014) and *acpPCSym_18* on days ten (2.44-fold, *p* = 0.012) and sixteen (2.08-fold, *p* = 0.020) (Fig. 5a,d,e). These three genes belong to two distinct LHC clades (Fig. 4), both *acpPCSym_15* and *acpPCSym_18* belong to Clade 1, while *acpPCSym_1:1* belongs to Clade 2. The largest fold change seen in these genes was a 2.44-fold increase in *acpPCSym_18* compared to control. For the remaining two *acpPCs* (*acpPCSym_5:1* and *acpPCSym_10:1*) which both belong to Clade 3b (Fig. 4) no significant changes in gene expression were detected (Fig. 5b,c). In addition to the five *acpPCs* the expression of the *psbA gene* was also determined. During the course of the experiment no significant differences in *psbA* expression between control and treatment conditions were found (Fig. 5f).

## Discussion

This study investigated the effect of increased temperatures on the expression of five *acpPC* genes in *Symbiodinium* under prolonged thermal stress of *A. aspera* and is the first to examine the expression of *acpPC* genes from different LHC Clades. The sixteen-day thermal regime was selected to enable sampling at temperatures leading up to, and inclusive of, a bleaching event and is the first experiment to investigate differential expression of integral antenna proteins in *Symbiodinium* within a coral host under thermal stress. Quantitative PCR was used to quantify the expression of five *acpPC* genes that are dispersed though out three clades of the chl *a*/*c* lineage of the LHC phylogeny (Fig. 4).

Over the course of the experiment temperature significantly effected *Symbiodinium* density and physiology. Symbiont cells decreased to approximately half the density in thermally stressed corals compared to control corals (Fig. 2a) as has been found in variety of other studies^4^,^35^,^38^. In addition chl *a* and chl *c* levels were elevated over the course of the experiment (Fig. 2b,c) in a manner seen before in this species (Gierz and Leggat, unpublished data)^35^. However, a statistical difference was only observed on day sixteen in chl *c* (Fig. 2c), this is consistent with other studies where an increase in chl pigments were observed^35^. In corals, heat-related increases in chl *a* have previously been recorded at low symbiont densities^39^,^40^, though in other experiments *Symbiodinium* pigmentation may be unchanged or decreased^41^,^42^. Increases in chl pigments have been attributed to repackaging of chls in the chloroplast membrane, with evidence that specific pigment-protein complexes may absorb more light at specific wavelengths^43^. In phytoplankton, chl *a*-specific absorption of different pigment-protein complexes from the same organism can be highly variable^43^. Therefore, it is possible that the increases in *Symbiodinium* pigments observed in heated corals may be attributed to alterations in the type of pigment-protein complexes expressed under thermal stress.

Imaging-pulse amplitude modulated (PAM) fluorometry analysis demonstrated that *Symbiodinium* cells exposed to elevated temperatures exhibited decreased photosynthetic efficiency (Fig. 3a), this is consistent with previous studies demonstrating the response of cells to elevated temperatures. Decreases in dark-adapted yield occurred throughout the experiment despite small changes in symbiont density, *F*_*v*_*/F*_*m*_ levels of ~0.00 were recorded on day sixteen despite cell density being approximately five hundred thousand per cm^2^, indicating cells were incapable of photosynthesis at the end of the stress period. Increased NPQ response in cells at days eight, ten and twelve (Fig. 3b) illustrates that the cells were dissipating excess light energy. However, this NPQ response was not present on day sixteen of thermal stress indicating that the symbionts had passed a threshold where photosynthetic processes were no longer functioning. Together, the photosynthetic efficiency results, *Symbiodinium* densities and changes to pigment levels, demonstrate that in this experiment *Symbiodinium* were subjected to the full range of temperatures that are seen in a bleaching event, with responses from initial thermal stress through to *Symbiodinium* expulsion. As such it is reasonable to conclude that *acpPC* expression patterns are representative of what would be seen in a natural bleaching event.

Expression of *acpPC* genes was found to vary in *Symbiodinium* cells throughout the experiment. Functionally little is known about the diversity of LHCs, for example whether complexes only associate with specific photosystems, are some more efficient at light capture or energy transfer, are others favoured for photoprotection or do some display increased stability under high temperatures. Characterisation of *Symbiodinium* *acpPCs* has shown that there is large diversity within the gene super-family^21^. Analysis of the *Symbiodinium* genome has provided more of an insight into the diversification of the LHC family^23^, reinforcing theories on gene duplication and deletion events leading to the current structure of the *Symbiodinium* genome. The complexity observed in the integral LHC family has been attributed to multiple rounds of intra- and inter-genic gene duplication events^20^,^23^. A large gene super-family encodes integral LHCs, and a significant level of sequence similarity has been detected between the protein complexes^21^,^23^. Phylogenetic analysis of LHCs and LHC-like protein super-families indicate that the ancestor is most likely a central group of two-helix stress-enhanced proteins that had previously evolved from a gene-duplication event of the high-light induced proteins of cyanobacteria^44^,^45^. However, based upon sequence divergence, it is reasonable to assume that different clades of *acpPC* may have different functions. As such, the differences in expression between those *acpPC* (Fig. 5a–e) from Clade 1 and 2 versus Clade 3b (Fig. 4), may be indicative of functional roles, with Clade 1 and 2 possibly being involved in stress response while those of Clade 3b are constitutively expressed under the conditions used here.

Some ways in which *acpPC* may functionally vary is in the binding of varied pigment ratios, specificity for association to photosystems and response to stress events. For example it has been found that a variety of *acpPC* transcripts are missing key chl and pigment binging residues^21^. In addition it is not clear to which photosystems different *acpPCs* bind. In green plants, ten highly conserved genes encoding chl *a*/*b* binding proteins have been identified, associated with photosystem I (PSI) are four pigment - protein complexes (encoded by genes *Lhca1, Lhca2, Lhca3* and *Lhca4*), and associated with PSII are six pigment – protein complexes (encoded by genes *Lhcb1, Lhcb2*, *Lhcb3*, *Lhcb4*, *Lhcb5*, *Lhcb6*)^20^. This can be contrasted to *Symbiodinium* where there is high sequence diversity coupled with high copy number, and as yet it is not clear which proteins bind to PSI or PSII^21^,^23^. It has been suggested that this sequence diversity allows for functional diversity such as, stress response^21^, attachment/dissociation^32^ and enhanced photoprotection^46^. As such, it will only be with the linkage of more transcriptome and genome studies, and the analysis of chl and accessory pigments binding residues, linked to functional studies, that we will be able to elucidate the reason for the expansion of this gene family in dinoflagellates.

Core photosystem genes, *psaA* and *psbA* have previously been investigated in *Symbiodinium* under thermal stress. Decreases of *psaA* and *psbA* are hypothesised to significantly impair the mechanisms associated with coping with thermal stress^47^. In this study, the expression of the *psbA* gene, which encodes the core PSII D1 protein, was also quantified (Fig. 5f). Over the course of the experiment *psbA* expression increased on days eight and ten (Fig. 5f) although expression in treatment samples was not statistically significant. However, on day sixteen, *psbA* expression decreased (Fig. 5f), potentially to reduce light absorption to limit the amount of energy captured under stress conditions as a photoprotective mechanism.

As in previous studies, investigating transcript abundance in *Symbiodinium* very small changes in gene expression were observed in this study. In the five *acpPC* genes quantified, the largest observed change was a 2.44 fold increase (*acpPCSym_18*) on day ten of the thermal stress experiment. In *Symbiodinium in hospite*, these small changes in transcripts have been observed previously^33^,^34^,^35^,^36^ and it is postulated that regulation is most likely post-translational and not at the transcriptional level^33^,^47^,^48^.

This study exploited a bleaching experiment to investigate the effect of thermal stress on photosynthetic genes. Quantitative PCR was used to determine the expression of five integral LHC genes. Three LHC genes (*acpPCSym_1:1*, *acpPCSym_15* and *acpPCSym_18*) were found to have increased expression over the duration of the experiment and interestingly, grouped in Clade 1 and Clade 2 of the LHC phylogeny. Additionally two LHC genes (*acpPCSym_5:1* and *acpPCSym_10:1*) grouped with Clade 3b did not exhibit differences in expression. Though transcriptional changes were detected, expression changes observed were less than 2.5 fold throughout the experiment. This is consistent with previous studies where small-scale changes in gene expression were also observed. Given that we currently do not know how the diverse range of LHCs are associated with the photosystems, both PSII and PSI, their specific functional roles, (e.g., light harvesting efficiency versus photoprotection), or the importance of *Symbiodinium* photosynthesis to the survival of corals, it is imperative that future research focuses on the specific roles of LHCs.

## Methods

### Thermal stress experimental design

Coral fragments (*n* = 312) were collected (under Great Barrier Reef Marine Park Authority permit G13/36402.1) from four different colonies of *Acropora aspera* (tan morph, approximately 78 from each colony) on the reef flat of Heron Island at low tide in May 2013. Heron Island colonies of *A. aspera* have been demonstrated to associate only with *Symbiodinium* clade C3^35^,^49^,^50^. Coral fragments were taken to the Heron Island Research Station and placed in a holding tub supplied with filtered water from the reef flat. Fragments were randomly selected and placed upright in racks. Nubbin racks were then transferred to eight 65 L replicate tanks and allowed to acclimate for five-days, at the end of this period tissue regrowth was observed on the cut section of the nubbins. Each of the replicate tanks were supplied with a flow of sand-filtered water pumped from the reef flat into two sump tanks, each sump tank then supplied water to four experimental tanks forming a semi-closed system.

The eight tanks were assigned to one of two treatments, control conditions (ambient temperature) and thermal-stress treatment conditions. The control conditions remained at the ambient seawater temperature (~24 °C) for the duration of the experiment (Fig. 1). Fluctuations observed in control conditions are natural daily temperature fluctuations (Fig. 1). The thermal-stress treatment temperature was increased by 0.7 °C for eleven days (25–32.3 °C), and then held at 33 °C for three days and then at 34 °C for a further three days (Fig. 1) to simulate a bleaching event. To achieve the required temperatures for the thermal treatment a three hundred Watt Eheim Jager (Eheim, Deisizou, Germany) heater was used in the heated sump as well as four 25 W Aqua One glass heaters. To ensure diurnal temperature variation in thermal treatment tanks the Eheim heater was turned off overnight to reflect natural fluctuations. Temperatures in each tank were recorded every 10 minutes with HOBO® temperature/alarm pendant data loggers (Onset, Massachusetts, USA). Light levels were monitored over the course of the experiment, every 10 minutes with Odyssey Photosynthetic Active Radiation (PAR) recorders (Dataflow Systems Limited, New Zealand).

### Imaging-PAM fluorometry

Imaging-PAM fluorometry (MAXI Imaging-PAM, Waltz, Effletrich, Germany) was used to measure photosynthetic efficiency of *Symbiodinium* within *A. aspera*. Imaging-PAM analysis was performed on the first day and every day from the third day following sunset. Nubbins were dark-adapted for twenty minutes prior to imaging-PAM analysis. Three replicate nubbins from each of the eight replicate tanks were designated for Imaging-PAM analysis and used throughout the experiment. Corals were measured in the same order at each imaging-PAM analysis. Dark-adapted yield and maximal fluorescence were determined using a weak pulse of light, followed by a saturating pulse of 2,700 μmol quanta m^−2^ s^−1^ of photosynthetically active radiation (PAR) for 800 ms. Induction recovery curves were used to examine the photosynthetic efficiency and ability of symbionts to recover from light stress throughout the experiment. The induction recovery analysis utilised fifteen pulses of saturating light (2,700 μmol quanta m^−2^ s^−1)^ with a constant actinic light (111 μmol quanta m^−2^ s^−1^) over a 5-minute period, followed by a dark recovery period of 14 min. Data from the induction recovery curve was used to determine photo-kinetic parameters, such as *F*_*v*_*/F*_*m*_ and NPQ. Following analysis, nubbins were returned to experimental tanks.

### Pigment quantification and *Symbiodinium* cell count

At 2 pm on days zero, eight, ten, twelve and sixteen, three replicate nubbins were taken from each replicate tank (*n* = 9–12) and were stripped of tissue using a Waterpik™ dental irrigator using seawater. On day zero the blastate was centrifuged at 3,076 g for 5 min immediately following tissue removal. On days eight, ten, twelve and sixteen the blastate was homogenised with an immersion blender for 5 s and centrifuged at 3,076 g for 3 min to pellet algal cells. Total pelleted cells were resuspended in 50 mL of seawater. Aliquots (1 mL) were taken for cell density approximation. The remaining cells were centrifuged at 3,076 g for 3 min to pellet cells and stored at −80 °C for chl *a* and *c* quantification. Chl was extracted in 90% acetone for 20 h in the dark at 4 °C and quantified using the equations of Jeffrey and Humphrey (1975)^51^. Cell number was determined using a Neubauer haemocytometer, with replicate cell counts performed (*n* = 5). Surface area of waterpiked nubbins was determined using the wax dipping method^52^.

### Gene expression analysis

At 2 pm on days five, eight, ten, twelve and sixteen, three replicate nubbins were taken from each replicate tank (total twelve nubbins per treatment) and snap-frozen in liquid nitrogen and stored at −80 °C for later mRNA isolation. Coral branches that had been snap-frozen in liquid nitrogen were crushed with a hydraulic press before transfer to a mortar chilled with liquid nitrogen and ground finely with a chilled pestle. The powder was then divided into cryotubes and stored at −80 °C. Messenger RNA was isolated from cells using the Dynabeads® mRNA DIRECT™ kit as per the protocol outlined in Leggat *et al.*^33^. Extracted mRNA was quantified spectrophotometrically using the Nucleic acid: RNA-40 setting on a NanoDrop-1000 (NanoDrop Technologies, Wilmington USA). DNase treatment of 0.1 μg of mRNA per reaction was carried out using RQ1 RNase-Free DNase (Promega) in a total volume of 9 μl. cDNA was reverse transcribed from DNase treated mRNA isolated from *Symbiodinium* cells for quantitative real-time-PCR (qRT-PCR). Reverse transcription was performed using the SuperScript™ III First-Strand Synthesis SuperMix for qRT-PCR (Invitrogen). The 2x RT Reaction Mix contains the following components oligo (dT)_20_ (2.5 μM), random hexamers (2.5 ng.μl^−1^), 10 mM MgCl_2_, and dNTPs. Template cDNA dilution series were prepared to optimize quantification accuracy. For analysis, cDNA was diluted 1:40 prior to use as a template in the qRT-PCR analysis. Quantitative RT-PCR was performed using a Rotor-Gene™ 6000 (Corbett Life Science, Australia). The qRT-PCR was performed in a final volume of 15 μl, containing 7.5 μl of GoTaq® qPCR Master Mix (Promega, USA), 4 μl of diluted template and gene specific primers (266 nM). qRT-PCR conditions were as follows: 95 °C for 2 min; 40 cycles of 95 °C for 15 s and 61 °C for 60 s. All qRT-PCR were followed by a melt curve analysis from 55 to 95 °C to ensure single product amplification (Supplementary Fig. S1). Each Rotor-Disc™-100 included three technical replicates for ten biological samples with three genes. Non-template controls for each primer set were performed in triplicate in each run.

Housekeeping genes used in our analysis were selected from previously established reference genes, which included Proliferating Cell Nuclear Antigen (PCNA)^36^, *cyclophin* (Cyc)^37^, *glyceraldehyde 3-phosphate dehydrogenase* (GAPDH)^37^, *S4 ribosomal protein* (Rp-S4)^37^ and *S-adenosyl-L-methionine synthetase* (SAM)^37^. Five *acpPC* (*acpPCSym_1:1*, *acpPCSym_5:1*, *acpPCSym_10:1*, *acpPCSym_15* and *acpPCSym_18*) *Symbiodinium* C3 primers were designed against *acpPC* sequences from an EST library^53^ obtained from the NCBI Genbank database (www.ncbi.nlm.nih.gov). Primers were designed for *acpPC* genes in *Symbiodinium* using the software DNASTAR Primer Select (Lasergene 11) (Table 1). The *psbA* primers were previously established for use in *Symbiodinium*^47^. Relative expression analysis was performed using qBASE plus 2.5 software (Biogazelle; http://www.biogazelle.com/products/qbasePLUS)^54^,^55^. Validation of housekeeping genes (expression stability and the optimum number of genes) in this experiment was performed using geNorm (qBASE plus)^54^,^55^.

### Data analyses

Statistics software package (SPSS Statistics v 22.0, IBM, USA) was used for all statistical analyses. A generalized linear model with ‘day’ and ‘treatment’ as main effects and ‘day × treatment’ as an interaction was used for pairwise comparisons of cell density, chl *a* and *c*, imaging-PAM and gene expression data. The sequential Bonferroni *post hoc* test was used to adjust for the false discovery rate (or type I error).

## Additional Information

**How to cite this article**: Gierz, S. L. *et al.* Integral Light-Harvesting Complex Expression In *Symbiodinium* Within The Coral *Acropora aspera* Under Thermal Stress. *Sci. Rep.* **6**, 25081; doi: 10.1038/srep25081 (2016).

## Supplementary Material

Supplementary Figure S1

## Figures and Tables

**Table 1:** Primer sequences and amplification efficiency used for quantitative PCR for *Symbiodinium.*

Gene name	Forward primer	Reverse primer	Reaction efficiency
*acpPCSym_1:1*	AGTGGAGTGAACCAGGAAGCAA	AACCAATCGCACCGACCAAGAG	1.05
*acpPCSym_5:1*	GGCGACTGCACCAAGGAGGACT	GAACACATCGGGCCAGAGCATACC	1.13
*acpPCSym_10:1*	GGAAACCCTAGCCGAGTGG	CTTGACATTTCCGAGAGCCTTCC	1.00
*acpPCSym_15*	GGGTGCCATTGAGTCTGTCC	TTAAGCCAAGGTCTCCCGCATTCT	0.96
*acpPCSym_18*	TCCCCTGGGCTTCTCTGATAC	GTTCTGCCACAAAGCCAATAGTT	1.01
*psbA*	TGCAGAAACTGCAGGAGATATTAGCC	TACTCCAAGGGCAGTGAACC	0.95
*Cyc*	ATGTGCCAGGGTGGAGACTT	CCTGTGTGCTTCAGGGTGAA	0.97
*GAPDH*	GGTGGTTGATGGCCAGAAGAT	CACCAGTGGATTCGCAAACA	1.06
*PCNA*	GAGTTTCAGAAGATTTGCCGAGAT	ACATTGCCACTGCCGAGGTC	1.00
*Rp-S4*	CCGCACAAACTGCGTGAGT	CGCTGCATGACGATCATCTT	0.99
*SAM*	GCCTACATTTGCCGACAGATG	AATGGCTTGGCAACACCAAT	1.03
