# Supplementary material for: Integral Light-Harvesting Complex Expression In Symbiodinium Within The Coral Acropora aspera Under Thermal Stress
Source: Sci Rep. 2016 Apr 27;6:25081. doi: 10.1038/srep25081 (PMC4846871; doi:10.1038/srep25081)
Supplement: Supplementary Figure S1 [file srep25081-s1.doc]

Integral Light-Harvesting Complex Expression In *Symbiodinium* Within The Coral *Acropora aspera* Under Thermal Stress

Sarah L. Gierz^a, b, *^, Benjamin R. Gordon^a, b^, William Leggat^a, b, c^

^a^ College of Public Health, Medical and Veterinary Sciences, James Cook University, Townsville, 4811, Australia

^b^ Comparative Genomics Centre, James Cook University, Townsville, 4811, Australia

^c^ ARC Centre of Excellence for Coral Reef Studies, James Cook University, Townsville, 4811, Australia

* sarah.gierz@my.jcu.edu.au


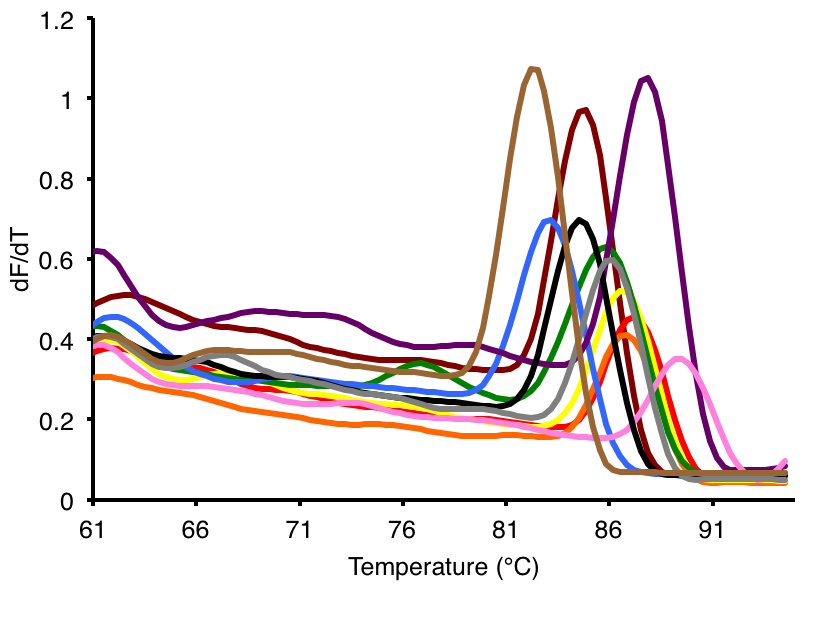


**Supplementary Figure S1.** Melt curve analysis of reaction products from qRT-PCR assay. Melt curve analysis for housekeeping genes, PCNA (maroon line), *cyc* (red line), *SAM* (orange line), *Rp-S4* (yellow line), GAPDH (green line). Melt curve analysis for genes of interest, acpPCSym_1:1 (blue line), acpPCSym_5:1 (purple line), acpPCSym_10:1 (pink line), acpPCSym_15 (black line), acpPCSym_18 (grey line) and *psbA* (brown line).
